# Integrating Epidemiology, Immunology, and Host Genetics for Controlling Peste des Petits Ruminants in Goats

**DOI:** 10.3390/v18091007

**Published:** 2026-09-13

**Authors:** Md Aminul Islam, Saifur Rahman, Md. Shafiqul Islam, Sharmin Aqter Rony, Asep Gunawan, Hari Om Pandey, Md. Taohidul Islam, A. K. M. Anisur Rahman, Md. Abu Hadi Noor Ali Khan, Julio Villena, Haruki Kitazawa, Muhammad Jasim Uddin

**Affiliations:** 1 Immunogenomics and Alternative Medicine (IAM) Laboratory, Department of Medicine, Faculty of Veterinary Science, Bangladesh Agricultural University, Mymensingh 2202, Bangladesh; 2 Department of Global Health and Social Medicine, Harvard Medical School, Boston, MA 02115, USA; 3 Department of Microbiology and Hygiene, Faculty of Veterinary Science, Bangladesh Agricultural University, Mymensingh 2202, Bangladesh; 4 Department of Parasitology, Faculty of Veterinary Science, Bangladesh Agricultural University, Mymensingh 2202, Bangladesh; 5 Department of Animal Production and Technology, Faculty of Animal Science, IPB University, Bogor 16680, Indonesia; 6 Department of Livestock Production and Management, Indian Veterinary Research Institute, Izatnagar, Bareilly 243122, UP, India; 7 Population Medicine and AMR Laboratory, Department of Medicine, Faculty of Veterinary Science, Bangladesh Agricultural University, Mymensingh 2202, Bangladesh; 8 Epidemiology and Preventive Medicine Laboratory, Department of Medicine, Faculty of Veterinary Science, Bangladesh Agricultural University, Mymensingh 2202, Bangladesh; 9 Department of Pathology, Faculty of Veterinary Science, Bangladesh Agricultural University, Mymensingh 2202, Bangladesh; 10 Laboratory Immunobiotechnology, Reference Centre for Lactobacilli (CERELA-CONICET), Tucuman 4000, Argentina; 11 Food and Feed Immunology Group, Laboratory of Animal Food Function, Graduate School of Agricultural Science, Tohoku University, Sendai 980-8572, Japan; 12 Livestock Immunology Unit, International Education and Research Center for Food and Agricultural Immunology (CFAI), Graduate School of Agricultural Science, Tohoku University, Sendai 980-8572, Japan; 13 School of Veterinary Medicine, Murdoch University, Murdoch Campus, Murdoch, WA 6150, Australia; 14 Centre for Biosecurity and One Health, Harry Butler Institute, Murdoch University, Murdoch Campus, Murdoch, WA 6150, Australia; 15 Centre for Animal Production and Health, Food Future Institute, Murdoch University, Murdoch Campus, Murdoch, WA 6150, Australia

**Keywords:** peste des petits ruminants virus, epidemiology, vaccination, host–virus interaction, host genetics, Black Bengal goats

## Abstract

Peste des petits ruminants (PPR) is a highly contagious transboundary viral disease of sheep and goats that causes major economic losses and livelihood disruption across Africa, the Middle East, and Asia. Goats are often more severely affected than sheep, particularly in endemic smallholder systems, where high turnover, nutritional stress, and limited veterinary infrastructure sustain viral transmission. Despite effective live attenuated vaccines and the ongoing FAO–WOAH Global Eradication Programme, PPR remains widely distributed, indicating that vaccination alone is insufficient without understanding of host, viral, and environmental determinants of disease persistence. This review synthesizes current knowledge on three interrelated pillars of PPR prevention and control in goats: epidemiology, immunology, and host genetics. We summarize the epidemiological drivers of PPR transmission and how these factors shape disease burden in endemic settings. We review the immunobiology of PPR virus (PPRV) infection and vaccination, focusing on innate antiviral sensing, adaptive immune protection, virus-induced immunosuppression, and field determinants of vaccine performance. Finally, we examine the emerging evidence for host genetic resilience to PPR, with emphasis on immunogenomic and transcriptomic findings and the relevance of indigenous breeds such as the Black Bengal goat as genomic resources. Current evidence suggests that genetic resilience to PPR is likely polygenic and remains insufficiently characterized, though genomic and transcriptomic tools offer opportunities to identify markers of reduced susceptibility, milder disease, or improved vaccine response. Sustainable control of PPR in goats will require integrated strategies combining mass vaccination, surveillance, improved husbandry, and host-focused immunogenomic research to strengthen herd resilience and accelerate progress toward global eradication.

## 1. Introduction

Peste des petits ruminants (PPR) is one of the most devastating transboundary viral diseases of small ruminants (sheep and goats) across large parts of Africa, the Middle East, and Asia [1,2,3]. The disease is caused by peste des petits ruminants virus (PPRV), a morbillivirus in the family Paramyxoviridae. It is characterized by fever, oculonasal discharge, stomatitis, diarrhea, bronchopneumonia, and, in severe outbreaks, high morbidity and mortality, particularly in immunologically naïve populations [2,4,5]. Because goats are often more severely affected than sheep and are central to the livelihoods of smallholder farmers in many endemic regions, PPR has major implications for household income, food security, women’s empowerment, and the resilience of mixed crop–livestock systems [6,7]. PPR is currently present in over 70 countries across Africa, the Middle East, and Asia, and its global economic impact is estimated at USD 1.5–2.1 billion annually [8,9,10], making the disease a priority target for international control and eradication efforts.

First described in Côte d’Ivoire in 1942 and initially considered a disease of West Africa, PPR is now established across large parts of Africa, the Middle East, and Asia [1,4,11,12]. Following the successful eradication of rinderpest, PPR was identified by the Food and Agriculture Organization of the United Nations (FAO) and the World Organisation for Animal Health (WOAH) as the next animal disease suitable for coordinated global eradication, with a target year of 2030 [8,9,10]. Several favorable features of PPR epidemiology support this goal, including the availability of effective live attenuated vaccines, the existence of a single serotype, and the absence of long-term carrier animals. Nevertheless, progress toward eradication has been uneven. PPR continues to circulate widely in endemic regions because transmission is sustained by complex interactions among host susceptibility, animal movement, vaccination gaps, husbandry practices, environmental stressors, and weaknesses in surveillance and veterinary service delivery [4,8,11]. These realities indicate that successful control of PPR requires more than vaccine availability alone; it demands a broader understanding of the biological and epidemiological factors that determine why some animals, flocks, and regions remain persistently vulnerable.

In goats, disease outcome after exposure to PPRV is not uniform. Clinical severity, mortality, and vaccine responsiveness vary according to age, maternal immunity, nutritional status, co-infections, management conditions, viral virulence, and likely host genetic background [5,13,14,15]. This variation places immunocompetence and genetic resistance or resilience at the center of current efforts to understand differential susceptibility to PPR and to optimize long-term control [6,13]. Immunocompetence influences whether exposure results in rapid viral clearance, effective vaccine-induced protection, or severe disease complicated by immunosuppression and secondary infection. Likewise, host genetic factors may contribute to between-breed and between-animal differences in susceptibility, inflammatory responses, survival, and vaccine responsiveness, although the molecular basis of such variation remains incompletely defined.

These questions are particularly relevant in South Asia, where goat production is both economically and socially important and where PPR remains endemic. In Bangladesh, the Black Bengal goat is the dominant indigenous breed and represents a valuable model for studying PPR under endemic village production systems. This breed is highly valued for prolificacy, adaptability, meat quality, and skin production, and it is often regarded as relatively hardy under low-input conditions [16,17]. At the same time, Black Bengal goats remain vulnerable to PPR, which continues to impose substantial direct and indirect losses through mortality, reduced productivity, treatment costs, and disruption of household income [5,18]. The growing availability of genomic resources for the Black Bengal goat offers an opportunity to investigate whether locally adapted breeds harbor genetic variation associated with disease resilience, vaccine responsiveness, or recovery following infection.

We use the Black Bengal goat throughout this review as an illustrative case study of an endemic, locally adapted breed with an expanding genomic toolkit, reflecting the authors’ regional expertise and the concentration of relevant genomic and epidemiological studies on this population. To state this explicitly: this review is intended as a global synthesis of PPR epidemiology, immunocompetence, and host genetics in goats, with Bangladesh and the Black Bengal goat serving as an in-depth regional case study rather than as the primary subject of the review. This emphasis is a deliberate focus rather than a claim that Black Bengal goats are broadly representative of goat populations worldwide. Comparable locally adapted breeds in other endemic regions—for example, the Red Sokoto and West African Dwarf goats in West Africa, the Small East African goat, and other indigenous South Asian breeds such as the Sirohi, Barbari, and Assam Hill goat—warrant similar investigation, and we do not intend the emphasis on Black Bengal goats to imply that this breed has broader or better-established resistance to PPR than other indigenous populations.

In this review, we synthesize current evidence on the prevention and control of PPR in goats by integrating three complementary perspectives: epidemiology, immunology, and host genetics. We first examine the epidemiological determinants of PPR transmission and persistence, with emphasis on host, viral, and environmental drivers in endemic systems. We then review current understanding of immune responses to PPRV infection and vaccination, including innate antiviral sensing, adaptive immunity, and PPRV-induced immunosuppression. Finally, we evaluate the emerging evidence for host genetic resistance or resilience to PPR, with particular attention to immunogenomic pathways, transcriptomic responses, and the relevance of Black Bengal goats and other indigenous breeds as resources for future marker discovery and breeding strategies. By linking these fields, we aim to provide a more integrated framework for designing sustainable PPR control strategies in goats and for identifying research priorities relevant to endemic production systems.

Despite substantial progress in understanding PPR virus (PPRV) biology and vaccine development, these advances have largely been investigated within separate disciplines, including epidemiology, immunology, and genomics. In reality, disease emergence, transmission, and control are governed by continuous interactions among these components. Epidemiological factors determine the intensity and pattern of viral exposure; host immunocompetence influences susceptibility, vaccine responsiveness, and viral clearance; and host genetic variation contributes to inter-animal differences in immune responses and clinical outcomes. Consequently, sustainable PPR control requires an integrated framework that combines field epidemiology, immunology, and host genomics rather than considering each discipline independently. Such a systems-based perspective is increasingly recognized as essential for supporting the FAO–WOAH Global Eradication Programme and for improving the effectiveness of vaccination, surveillance, and breeding strategies in endemic production systems [4,8,9,19].

## 2. Search Strategy and Evidence Synthesis

This study was conducted as a state-of-the-art narrative review to critically synthesize current evidence on the epidemiology, immunology, and host genetics of PPR in goats [20]. Unlike a systematic review, no predefined PRISMA protocol, formal risk-of-bias assessment, or quantitative meta-analysis was undertaken. Instead, the objective was to provide an integrative and interdisciplinary synthesis of the available evidence and identify key research gaps [20].

A structured literature search was performed primarily in PubMed, supplemented by backward citation tracking of relevant articles and authoritative grey literature from the World Organisation for Animal Health (WOAH) and the Food and Agriculture Organization of the United Nations (FAO). Searches combined controlled vocabulary and free-text terms across nine concept blocks: (i) PPR epidemiology; (ii) risk factors and transmission; (iii) disease frequency, distribution, and surveillance; (iv) PPR vaccines and vaccination; (v) immunocompetence and vaccine-induced immunity; (vi) Black Bengal goats and caprine genomics; (vii) host–PPRV interactions, transcriptomics, and immunogenomics; (viii) genetic resistance, resilience, and breeding approaches; and (ix) surveillance, policy, and eradication strategies (Supplementary Table S1). The complete Boolean search strings used for each concept block, together with the database searched, search execution dates, and the number of records retrieved per block, are provided in Supplementary Table S1.

Studies were eligible for inclusion if they (i) reported original data, a peer-reviewed synthesis, or authoritative technical guidance on PPR epidemiology, immunopathology, immunocompetence, vaccination, or host genetics/genomics in goats or closely related small ruminants; (ii) were published in English; and (iii) appeared in a peer-reviewed journal or, for surveillance and policy information, in an official WOAH or FAO technical document. Studies were excluded if they (i) addressed morbillivirus infection exclusively in species without demonstrated relevance to goat PPR; (ii) were conference abstracts, non-peer-reviewed preprints, or opinion pieces lacking primary or synthesized data; or (iii) were not accessible in full text. Literature published from 2000 onward was preferentially included to reflect contemporary diagnostic, vaccine, and genomic evidence, while landmark earlier studies (e.g., original descriptions of PPRV lineages and pathogenesis) were retained for historical and conceptual context regardless of publication date. Studies addressing PPR exclusively in sheep, general livestock immunology without a PPRV- or morbillivirus-specific component, or broad caprine genomics unrelated to immune function or disease traits were excluded unless they provided mechanistic or comparative insight not otherwise available from goat-specific literature, in which case they were retained but explicitly flagged in the text as extrapolated rather than direct goat-PPR evidence.

Because direct goat-specific evidence is not uniformly available across all topics covered in this review, we applied an explicit evidentiary hierarchy when synthesizing findings and judging their strength: highest weight was given to studies conducted directly in goats naturally or experimentally infected with PPRV; where goat-specific data were limited, we drew on evidence from mixed sheep-goat populations, other morbillivirus systems (e.g., measles virus, rinderpest virus), or general livestock immunology. Such instances are explicitly flagged in the text with qualifying language (e.g., “evidence from mixed sheep-goat studies suggests…”, “by analogy with other morbilliviruses…”, “direct goat-specific evidence remains limited”) rather than being presented as direct goat-specific findings, so that readers can assess the strength and directness of the underlying evidence for each claim.

The initial PubMed search across the nine concept blocks (Supplementary Table S1) retrieved 4822 records. After removal of duplicates, 2101 records were screened by title and abstract against the eligibility criteria described above, of which 412 were assessed in full text. 114 records met the eligibility criteria, and a further 9 records were added through backward citation tracking and WOAH/FAO grey literature; finally, 81 sources are cited in the final synthesis. Screening and eligibility decisions were made by the corresponding author and cross-checked by co-authors. Screening was performed by a single reviewer (the corresponding author) with periodic cross-checking of inclusion decisions by co-authors with relevant subject-matter expertise (epidemiology, immunology, and genomics); disagreements were resolved by discussion and consensus among the authorship team, and no formal inter-rater reliability statistic was calculated, consistent with the narrative (rather than systematic) review design. Because this was conducted as a narrative rather than a systematic review, dual independent screening, a PRISMA flow diagram, and a formal risk-of-bias assessment were not undertaken. Evidence was synthesized narratively to integrate findings across epidemiology, immunology, genomics, and disease-control research, with emphasis on current knowledge, emerging evidence, and future research priorities. The literature search was conducted from 1 January 2020 through 26 August 2026.

We acknowledge that reliance on PubMed as the primary database, while appropriate given its strong coverage of the virological and biomedical literature on PPR, may have resulted in incomplete capture of veterinary-specific and regional literature indexed mainly in databases such as CAB Abstracts, Web of Science, or Scopus, or published in regional veterinary journals not indexed in PubMed. This is a recognized limitation of single-database narrative reviews and may be particularly relevant for field-level epidemiological studies from South Asia and Africa. Backward citation tracking and inclusion of WOAH and FAO grey literature were used to partially mitigate this limitation, but a future systematic review on this topic would benefit from multi-database searching (e.g., PubMed, CAB Abstracts, Web of Science, Scopus) and formal PRISMA methodology.

To support more critical, comparative synthesis across the evidence base, Table 1 summarizes representative epidemiological, immunological, and genetic studies discussed throughout this review, together with their design, key findings, and principal limitations, and explicitly distinguishes established findings from proposed mechanisms.

## 3. Epidemiology of PPR in Goats

### 3.1. Disease Frequency and Global Occurrence

Peste des petits ruminants (PPR) remains endemic across much of sub-Saharan and North Africa, the Middle East, and large parts of Asia, where it causes recurrent outbreaks in goats and sheep and continues to constrain small-ruminant production and trade [1]. In quantitative terms, PPR is now present in more than 70 countries, which are collectively home to over 1.7 billion sheep and goats, and its annual global economic impact is estimated at USD 1.5–2.1 billion [9,10]. Up-to-date, interactive maps of country-level PPR occurrence and official disease-free status are maintained by WOAH through its World Animal Health Information System (WAHIS); we point readers to this resource rather than reproducing a static map here, since WAHIS data are revised continuously as new outbreaks and freedom declarations are reported. According to WOAH and FAO, PPR is currently one of the priority transboundary animal diseases targeted for global eradication, yet its epidemiological burden remains substantial because of persistent endemic circulation, incomplete vaccination coverage, transboundary livestock movement, and uneven surveillance capacity across affected regions [11,35,36]. The frequency of PPR is usually described using seroprevalence, outbreak incidence, morbidity, case fatality, and spatiotemporal recurrence of outbreaks. However, direct comparison among studies is difficult because estimates vary with study design, diagnostic method, vaccination history, and whether cases are clinically suspected or laboratory confirmed.

At the population level, serological surveys and meta-analyses indicate widespread exposure to PPRV in endemic regions, although prevalence varies markedly across countries, production systems, and age groups. In Bangladesh, a recent meta-analysis estimated a pooled prevalence of approximately 31% in goats, highlighting sustained virus circulation in the national small-ruminant population [12]. Nevertheless, prevalence estimates based solely on serology should be interpreted cautiously because they reflect cumulative exposure and may also be influenced by vaccination status, assay performance, and local outbreak history. For prevention planning, therefore, epidemiological interpretation should integrate serology with clinical surveillance, laboratory confirmation, animal movement data, and vaccination coverage.

### 3.2. Distribution of PPR in Goat Populations

#### 3.2.1. Host and Demographic Distribution

PPRV exhibits marked tropism for lymphoid and epithelial tissues, particularly the respiratory and gastrointestinal mucosa and associated lymphoid organs. Following infection through the respiratory route, the virus initially replicates in the nasopharyngeal epithelium and regional lymphoid tissues before disseminating systemically, leading to immunosuppression, mucosal damage, and high viral shedding during acute disease [3,4]. This pathobiology is central to the epidemiology of PPR because it facilitates efficient transmission in settings where susceptible goats are housed, grazed, or traded in close contact.

Age is among the most consistent host-level determinants of PPR occurrence. Young goats are generally more susceptible to clinical disease than adults, particularly after the waning of maternally derived antibodies and before the development of robust active immunity. In many endemic settings, the highest burden is observed in animals between approximately 4 and 12 months of age, although the exact age window varies with maternal immunity, management, and vaccination practices [15,37]. In Bangladesh, Black Bengal goats aged 7–12 months have repeatedly been reported to be at increased risk of PPR compared with younger kids, likely reflecting declining maternal antibody protection and increasing exposure after weaning and mixing [5].

The role of sex in PPR epidemiology is less consistent. Several field studies have reported higher seroprevalence or case occurrence in females [38], often attributed to longer retention of breeding females within flocks, pregnancy- and lactation-associated stress, or sex-related management differences rather than intrinsic biological susceptibility alone. In contrast, other studies have found no significant sex effect or even a higher occurrence in males. Overall, sex-associated differences in PPR risk appear to be context-dependent and likely confounded by husbandry practices, age structure, and production purpose; therefore, sex should be interpreted as an epidemiological marker rather than a universally reproducible biological determinant.

Breed and genotype may also influence disease susceptibility, although the evidence remains incomplete. Goats are generally more severely affected than sheep, but within goats, inter-breed variation in clinical outcome and serological response has been described in Africa and South Asia [15,29]. Field observations suggest that susceptibility to PPR varies among goat breeds, although the underlying mechanisms remain incompletely understood. Being an indigenous breed, Black Bengal goats have frequently been reported to experience lower morbidity or mortality than some exotic or crossbred populations under endemic conditions [22]. These comparisons have typically involved Black Bengal goats alongside exotic dairy or meat breeds and their crosses raised under the same smallholder or peri-urban management systems in Bangladesh, rather than formal multi-breed field trials with standardized exposure; comparable field-level breed differences, involving the Assam Hill goat and other locally adapted populations, are discussed further in Section 5.4. However, these observations should be interpreted cautiously because differences in management, nutrition, vaccination history, and environmental adaptation may also contribute to the observed variation. Consequently, current evidence supports the existence of breed-associated resilience rather than genetically confirmed resistance, highlighting the need for well-designed genomic association studies.

Concurrent disease and poor physiological status further shape PPR expression at the individual and flock levels. Morbilliviruses induce profound but transient immunosuppression through lymphoid depletion and leukocyte dysfunction, predisposing infected goats to secondary bacterial pneumonia, enteric complications, and mixed infections that amplify mortality [3,4]. Field reports have described co-infections involving PPRV and capripoxviruses, orf virus, adenoviruses, and other pathogens, although the epidemiological importance of specific combinations varies among settings. Malnutrition, parasitism, late pregnancy, transport stress, and poor housing likely exacerbate disease severity by compromising host immunocompetence.

#### 3.2.2. Spatial Distribution

PPRV is genetically classified into four lineages (I–IV) based primarily on partial nucleoprotein (N) or fusion (F) gene sequences. Although these lineages do not constitute distinct serotypes, tracking their geographic distribution is epidemiologically important for tracing transmission pathways, reconstructing outbreak origins, and monitoring the changing geography of circulating strains. Historically, lineage I and II viruses were associated mainly with western and central Africa, lineage III with eastern Africa and parts of the Arabian Peninsula, and lineage IV with South Asia, the Middle East, and later broad expansion into Africa. In recent years, lineage IV has become the dominant globally circulating lineage and has displaced or co-circulated with older regional lineages in multiple settings, emphasizing the dynamic transboundary nature of PPRV spread [3,4].

The current global distribution of PPR is shaped by a combination of livestock mobility, informal trade, pastoral and transhumant systems, cross-border mixing, and gaps in vaccination coverage. Recent incursions into previously unaffected areas illustrate this risk in concrete, quantitative terms: PPR was detected for the first time in Greece and Romania in July 2024, with 47 outbreaks and over 2000 reported cases in Greece and 56 outbreaks and over 5000 reported cases in Romania by early August 2024, followed by further detection in Bulgaria in November 2024 [39], countries with no prior history of the disease. In South Asia, PPR was first recognized in India in the late 1980s and subsequently reported in Pakistan, Bangladesh, Nepal, Bhutan, Afghanistan, and China [40]. Molecular studies indicate that Bangladeshi viruses belong to lineage IV and are closely related to strains circulating in neighboring South Asian countries, consistent with repeated regional connectivity and cross-border viral movement [22].

#### 3.2.3. Temporal Distribution

PPR is not uniformly distributed throughout the year. In endemic settings, outbreaks often show seasonal clustering linked to animal movement, congregation around markets or communal grazing areas, nutritional stress, and climatic conditions that influence contact structure rather than long-term environmental persistence of the virus [41]. In Bangladesh, higher occurrence has frequently been reported during the cooler months and around periods of increased animal movement, although seasonal peaks vary among studies and production systems [21,22]. Similar seasonal patterns have been described elsewhere in Africa and Asia, often coinciding with transhumance, breeding cycles, feed scarcity, or festival-associated trade.

The seasonal epidemiology of PPR should therefore be interpreted primarily through changes in host behavior and contact networks rather than through climate alone. For example, the congregation of susceptible goats at watering points, grazing fields, and live-animal markets can substantially increase effective contact rates. Likewise, seasonal kidding may transiently increase the proportion of immunologically naïve animals within flocks, thereby facilitating transmission.

### 3.3. Determinants of PPR Occurrence and Spread

#### 3.3.1. Pathogen-Related Factors

PPR is caused by peste des petits ruminants virus (PPRV), an enveloped, non-segmented, negative-sense single-stranded RNA virus of the genus Morbillivirus within the family Paramyxoviridae [1,4]. The virus has a single serotype but is genetically classified into four lineages (I–IV) based primarily on partial nucleoprotein (N) or fusion (F) gene sequences. Although these lineages do not constitute distinct serotypes, lineage classification is epidemiologically important for tracing virus movement, reconstructing transmission pathways, and understanding the changing geography of PPRV circulation.

Among the four lineages, lineage IV has achieved the broadest contemporary distribution and now predominates across South Asia, China, much of the Middle East, and increasing parts of Africa [4,11]. Available sequence data from Bangladesh indicate circulation of lineage IV viruses closely related to regional strains from neighboring countries [22]. More extensive whole-genome sequencing of Bangladeshi isolates, including viruses from Black Bengal goats, would improve understanding of viral introduction routes, local persistence, and potential microevolution under endemic field conditions. Although current vaccines provide cross-lineage protection because PPRV is serologically monotypic, genomic surveillance remains essential for molecular epidemiology, outbreak tracing, and evaluation of vaccine–field strain relationships.

#### 3.3.2. Host, Management, and Environmental Risk Factors

PPR occurrence is driven by the interaction of host susceptibility, infection pressure, and management context. Across endemic regions, repeatedly identified risk factors include young age, lack of vaccination, introduction of new animals without quarantine, flock mixing at markets or communal grazing sites, transboundary or seasonal movement, high local animal density, poor biosecurity, and nutritional or physiological stress [1,3,35]. Conversely, protective factors include effective vaccination, flock immunity above the local transmission threshold, isolation of clinically affected animals, quarantine of purchased stock, reduced mixing during outbreaks, and adequate nutrition and general health management.

Environmental conditions can influence PPR epidemiology indirectly by shaping host aggregation and movement. In pastoral and agro-pastoral systems, drought, feed scarcity, and seasonal rainfall patterns alter grazing routes, watering-point use, and market attendance, thereby modifying contact rates among flocks. Similarly, peri-urban intensification and commercial live-animal trade can create high-density contact networks that facilitate rapid dissemination of infection. These factors do not act independently; rather, they interact with vaccination gaps and surveillance weaknesses to determine whether virus introduction results in sporadic cases, localized outbreaks, or sustained endemic transmission.

### 3.4. Transmission Dynamics in Goats

PPRV is transmitted primarily by close contact through respiratory secretions and other excretions from infected animals. Shedding is highest during the acute phase of disease, when goats excrete virus in ocular, nasal, and oral secretions as well as in feces, thereby facilitating transmission within households, communal grazing systems, transport chains, and livestock markets [3,4]. Indirect transmission via contaminated fomites is possible over short time frames but is considered less important than direct contact because PPRV is an enveloped virus with limited environmental persistence under field conditions.

The incubation period of PPR is typically 4–6 days under experimental and field conditions, with a broader range of approximately 3–10 days depending on infectious dose, route of exposure, and host factors [8]. Infected goats may begin shedding virus shortly before or around the onset of clinical signs, and infectiousness generally peaks during the acute febrile and mucosal phase. Because clinical expression can vary from acute fatal disease to milder infection, especially in partially immune populations, subclinical or less obvious infections may still contribute to local transmission if surveillance relies solely on severe clinical presentation.

At the population level, the transmission potential of PPR is often summarized using the basic reproduction number (R_0_) or the effective reproduction number (R_t_). Published estimates vary by setting and model structure, but available evidence indicates that PPR can spread rapidly in susceptible goat populations, particularly where flock mixing and animal movement are intense, and vaccination coverage is inadequate. This has important implications for herd immunity. In practice, the vaccination coverage required for control is usually higher than the theoretical herd-immunity threshold calculated from simple (R_0_)-based formulas because field effectiveness is reduced by heterogeneous coverage, imperfect campaign timing, population turnover, maternal antibody interference, and persistent pockets of unvaccinated animals. For this reason, PPR eradication programmes emphasize repeated strategic vaccination, movement-sensitive surveillance, and risk-based targeting of high-connectivity populations rather than reliance on a single universal coverage threshold [8,42]. In practice, vaccination effectiveness is most often assessed through post-vaccination sero-monitoring, in which a sample of vaccinated animals is tested for neutralizing or ELISA-detectable antibodies at a defined interval after immunization, and the proportion seroconverting is compared against a pre-specified population-immunity target. For example, post-vaccination sero-monitoring programmes for PPR in Karnataka, India used a target population-immunity threshold of approximately 70% to gauge progress toward interrupting transmission (discussed further with the underlying seroconversion data in Section 6.1) [33,43].

From an epidemiological perspective, the most important amplifiers of PPR transmission in goats are not only viral shedding and susceptibility, but also the structure of contact networks among flocks. Livestock markets, communal grazing lands, seasonal migration routes, and the introduction of replacement stock connect otherwise separate goat populations and can sustain transmission across districts or national borders. Therefore, integrating classical outbreak investigation with molecular epidemiology, animal movement analysis, and vaccination data is essential for identifying transmission hotspots and optimizing control strategies in endemic settings.

The epidemiological patterns of PPR cannot be explained solely by viral circulation or animal movement. Whether exposure results in subclinical infection, severe disease, or effective immune protection depends on the interaction between viral characteristics and the immunological competence of the host. Factors such as age, nutritional status, concurrent parasitic or bacterial infections, physiological stress, maternal antibody interference, and vaccination history influence the quality of innate and adaptive immune responses and, consequently, alter transmission dynamics at both the individual and population levels. Thus, host immunocompetence represents the biological link through which epidemiological risk factors ultimately translate into disease outcome and herd immunity.

## 4. Immunology of PPR in Goats

Immunocompetence refers to the capacity of an animal to mount effective innate and adaptive immune responses to infection or vaccination while limiting immunopathology. In goats, immunocompetence is a key determinant of susceptibility to PPRV, clinical outcome, vaccine responsiveness, and recovery after infection [44]. Because no specific antiviral therapy is available for PPR, host immune competence remains central to both natural resistance and successful prevention through vaccination. At the population level, immunocompetence is shaped by a combination of host genetics, age, maternal immunity, nutritional status, concurrent infections, stress, microbiome composition, and vaccine history. These interacting factors are especially relevant in endemic smallholder systems, where repeated pathogen exposure, undernutrition, and management stress may compromise immune performance and alter vaccine effectiveness.

For clarity, we use immunocompetence, vaccine responsiveness, general health status, and herd immunity as related but distinct terms throughout this review. Immunocompetence describes an individual animal’s intrinsic capacity to mount effective innate and adaptive immune responses. Vaccine responsiveness is one manifestation of immunocompetence, referring specifically to the magnitude and durability of the immune response elicited by vaccination rather than natural infection. General health status (nutrition, concurrent infection, and physiological stress) is an input that modifies immunocompetence rather than being synonymous with it. Herd immunity, by contrast, is a population-level epidemiological concept describing the proportion of immune individuals required to interrupt transmission; it is a downstream consequence of individual immunocompetence and vaccination coverage rather than an individual trait, and we avoid using “population immunocompetence” as a substitute for it.

### 4.1. Innate Immune Responses to PPRV Infection and Vaccination

The innate immune response provides the first layer of defense against PPRV and is initiated within hours of infection or vaccination. As with other morbilliviruses, PPRV enters susceptible cells primarily through receptor-mediated attachment and fusion. The signaling lymphocyte activation molecule (SLAM/CD150) is the major receptor on immune cells, whereas nectin-4 is important for epithelial tropism and viral shedding from mucosal surfaces [3,4,45]. It is important to distinguish these viral entry receptors from pattern-recognition receptors (PRRs): SLAM and nectin-4 mediate cell entry, whereas PRRs detect viral nucleic acids and initiate antiviral signaling.

Following infection or exposure to live attenuated vaccine virus, viral RNA is sensed by host PRRs, particularly endosomal Toll-like receptors and cytosolic RNA sensors such as retinoic acid-inducible gene-I (RIG-I)-like receptors and melanoma differentiation-associated protein 5 (MDA5). Activation of these pathways induces type I interferons (IFN-I), interferon-stimulated genes, inflammatory mediators, and antiviral effector mechanisms that restrict viral replication and help shape subsequent adaptive immunity [23,46,47]. Although the PRR landscape of PPRV infection in goats is not yet fully defined, transcriptomic and experimental studies indicate that early host responses include induction of interferon-related pathways, cytokine signaling, and inflammatory gene networks in infected epithelial and lymphoid tissues [23,48].

PPRV infection is associated with a marked but complex cytokine response. Increased expression of IFN-β, IFN-γ, IL-1β, IL-6, IL-8, IL-10, and IL-12 has been reported in infected tissues or peripheral blood, although the magnitude and timing vary with viral strain, tissue compartment, disease stage, and host species [3,23,49]. Histopathological studies have also demonstrated enhanced expression of inducible nitric oxide synthase and pro-inflammatory mediators in oral and respiratory mucosa, consistent with local innate activation at major sites of viral replication. However, PPRV is not simply a trigger of innate immunity; like other morbilliviruses, it also encodes immune-evasion mechanisms that dampen antiviral signaling. In particular, the viral V protein antagonizes interferon induction and signaling through interactions with molecules in the RIG-I/MDA5 and JAK-STAT pathways, thereby facilitating viral replication and contributing to immunosuppression [4,50].

From the perspective of host resistance, the timing and magnitude of innate responses are likely critical. A rapid and coordinated early interferon response may limit viral spread, reduce tissue damage, and support efficient priming of adaptive immunity, whereas delayed or dysregulated innate activation may favor severe disease. This has practical relevance for both natural infection and vaccination, because variability in innate responsiveness may influence the quality and durability of protective immunity. It also supports the view that genes involved in antiviral sensing, interferon signaling, and inflammatory regulation are plausible candidates for genetic resistance or resilience to PPR in goats.

### 4.2. Adaptive Immunity to PPRV and Vaccine-Induced Protection

Protective immunity to PPRV depends on the coordinated development of humoral and cell-mediated responses. Following infection or vaccination, antigen-presenting cells activate CD4+ T cells, which provide help for B-cell differentiation and antibody production, and CD8+ T cells, which contribute to viral clearance through cytotoxic activity and cytokine secretion. Neutralizing antibodies directed mainly against the viral hemagglutinin (H) and fusion (F) glycoproteins are considered the principal correlate of protection, whereas cell-mediated immunity is important for virus clearance, memory formation, and possibly cross-protection among morbilliviruses [4,51,52]. Collectively, these studies indicate that PPRV induces a coordinated antiviral response characterized by activation of interferon signaling, inflammatory cytokines, and adaptive immune pathways. Although individual studies differ in experimental design and host species, they consistently demonstrate that successful viral clearance depends on rapid innate immune activation followed by robust T- and B-cell responses.

Among PPRV proteins, the H and F glycoproteins are the major targets of protective neutralizing antibody responses. By contrast, the nucleoprotein (N), although highly abundant and strongly immunogenic, is not a major target of virus-neutralizing antibodies. Instead, N appears to contribute predominantly to T-cell responses and is useful as an immunodiagnostic antigen because of its strong sero-reactivity [3,23]. Experimental studies have shown that recombinant vaccines expressing H or F can induce both antibody and T-cell responses, supporting the central role of these glycoproteins in protective immunity [52]. Antibody-dependent cellular cytotoxicity and natural killer cell-mediated mechanisms may also contribute to protection, although their role in natural PPRV infection remains less well characterized than neutralizing antibody responses [52].

Live attenuated PPRV vaccines induce robust adaptive immunity in both sheep and goats and remain the cornerstone of global control programmes. Classical vaccine strains such as Nigeria 75/1 and Sungri/96 generate seroconversion in most vaccinated animals and are capable of inducing long-lasting protection after a single dose under controlled conditions [4,51]. Recent evidence confirms that vaccination with Nigeria 75/1 elicits strong humoral responses and durable immunity in adult sheep and goats, with seroconversion detectable by approximately 3 weeks after immunization and protection persisting for several years [24,53]. These immunogenicity findings derive largely from controlled experimental studies in adult, mixed sheep-goat cohorts; goat-specific data under field conditions and in young, recently weaned animals (the age group at greatest clinical risk) are comparatively limited, and vaccine-performance claims below should be read with this distinction in mind. Nevertheless, vaccine performance under field conditions is influenced by maternal antibody interference, nutritional stress, concurrent disease, cold-chain integrity, and the age structure of the flock. Thus, immunocompetence should be considered not only as a host trait but also as a field-level determinant of vaccine effectiveness.

### 4.3. PPRV-Induced Immunosuppression and Immunocompetence

A defining feature of morbillivirus infections is their capacity to induce profound but often transient immunosuppression, and PPRV is no exception. PPRV is highly lymphotropic and infects immune cells expressing SLAM/CD150, resulting in lymphoid depletion, leukopenia, impaired lymphocyte proliferation, and increased susceptibility to secondary infections [3,4,54]. Clinically, this immunosuppression is reflected in the frequent occurrence of severe bacterial pneumonia, enteric complications, and prolonged weakness in affected animals. At the tissue level, necrosis of lymphoid organs, depletion of lymphocytes, and disruption of mucosal immunity contribute to both disease severity and increased shedding.

Mechanistically, PPRV-induced immunosuppression is multifactorial. Proposed mechanisms include direct infection and depletion of leukocytes, inhibition of interferon responses, altered cytokine signaling, suppression of lymphocyte proliferation, and immune dysfunction mediated by viral structural and non-structural proteins. By analogy with other morbilliviruses, studies in measles virus and rinderpest virus have shown that viral glycoproteins and nucleoproteins can impair lymphocyte activation and proliferation, and analogous effects have been demonstrated for PPRV, including an inhibitory role of the nucleoprotein in lymphoproliferation [55,56], though direct goat-specific mechanistic data remain more limited than for the better-studied measles and rinderpest systems. In addition, the PPRV V protein is an established interferon antagonist that interferes with antiviral signaling and likely contributes to the depth of immunosuppression during acute infection [50].

This immunosuppressive phase has important epidemiological and clinical consequences. It increases the likelihood of secondary bacterial and parasitic complications, may worsen case fatality in nutritionally stressed or heavily parasitized goats, and can obscure the interpretation of immune biomarkers during acute disease. That live attenuated vaccines cause milder disease than virulent field strains while still inducing protective immunity is a general property of attenuated vaccination shared with many other pathogens, and is not by itself a distinguishing feature of PPR. What is specifically relevant for PPR is that the pathology removed by attenuation is not only overt clinical disease but also the profound, transient lymphoid depletion and immunosuppression that characterize virulent PPRV infection: live attenuated vaccines may cause transient leukopenia or short-lived immune perturbation, but they do not reproduce this immunosuppressive phenotype [4]. Because PPRV-induced immunosuppression is itself a major driver of secondary bacterial and parasitic complications and of case fatality in the field, the absence of this specific effect after vaccination is a clinically meaningful safety property, distinct from simple protection against overt PPR signs, and helps explain why live attenuated vaccines remain safe even in nutritionally stressed or concurrently infected animals.

### 4.4. Vaccination as a Tool to Enhance Population Immunity

Section 4.1, Section 4.2 and Section 4.3 establish three points that bear directly on the vaccination strategies discussed below. First, the hemagglutinin (H) and fusion (F) glycoproteins are the principal targets of neutralizing antibody responses (Section 4.2), which is why current and experimental PPR vaccines are designed around eliciting immunity to these two proteins specifically, rather than to the more abundant but non-neutralizing nucleoprotein. Second, the speed and quality of the early innate interferon response shapes how efficiently vaccine antigen is processed and how robust the resulting adaptive response becomes (Section 4.1), offering one plausible biological explanation for inter-animal variation in vaccine “take” that is not attributable to antigen dose or cold-chain handling alone. Third, live attenuated PPR vaccines are effective specifically because they elicit this protective antibody and cellular response without reproducing the profound immunosuppression that characterizes virulent field infection (Section 4.3); this distinction underlies both their efficacy and their field safety, including in nutritionally stressed or concurrently infected animals. The remainder of this section examines how these principles translate into vaccine performance and field effectiveness in practice.

Vaccination is the most effective intervention for increasing population immunity against PPR and remains the foundation of the FAO–WOAH eradication strategy. In endemic settings, especially where culling-based control is impractical, mass vaccination reduces the pool of susceptible animals, limits viral transmission, and indirectly protects young stock through reduced infection pressure. Live attenuated vaccines based on strains such as Nigeria 75/1 and Sungri/96 have shown high efficacy and broad cross-lineage protection because PPRV is serologically monotypic [4,51]. Under current WOAH recommendations, animals are typically vaccinated from approximately 3–4 months of age or after waning of maternal antibodies, although optimal timing depends on local epidemiology, maternal immunity, and vaccination strategy.

Field effectiveness, however, depends on more than vaccine antigenicity. In practice, vaccine impact is shaped by campaign coverage, maintenance of the cold chain, animal movement, age at vaccination, nutritional status, and the immunological competence of the target population. This is particularly relevant in Bangladesh, where conventional live attenuated PPR vaccines have been used for many years and where thermostable vaccine formulations were developed to address cold-chain constraints under rural field conditions. Experimental and field studies from Bangladesh have shown that both conventional and thermostable formulations can induce serological responses in goats, but real-world protection is likely influenced by storage conditions, timing of vaccination, and the health status of vaccinated animals [25,26]. These considerations underscore that vaccine failure in the field does not necessarily imply antigenic mismatch; it may also reflect operational failures or host-related limitations in immune responsiveness.

PPR vaccine platforms differ substantially in their current development and field-use status, and these distinctions are important for interpreting the literature. *Conventional live attenuated vaccines* (e.g., Nigeria 75/1, Sungri/96) are in routine field use worldwide and form the backbone of the FAO–WOAH eradication programme. *Thermostable formulations* of these same live attenuated strains have been developed and field-tested, including in Bangladesh, to address cold-chain constraints; they are increasingly deployed in some endemic countries but are not yet universally available. By contrast, *recombinant, viral-vectored, and DIVA-compatible candidates* (including recombinant capripoxvirus-, adenovirus-, and reverse-genetics-based platforms) remain at the experimental or pre-clinical stage. These platforms have shown promise in laboratory and small-scale animal studies, particularly because several would permit differentiation of infected from vaccinated animals (DIVA), a feature not offered by conventional live attenuated vaccines [34]. However, none is currently licensed or deployed in routine field vaccination campaigns, and substantial regulatory, manufacturing, and cost barriers remain before field adoption is feasible.

### 4.5. Nutrition, Gut–Mucosal Immunity, and Immunobiotic Support

Nutritional status is a major but often underappreciated determinant of immunocompetence in goats. Adequate dietary protein, energy, vitamins, and trace minerals are essential for maintaining epithelial barrier function, leukocyte activity, antibody production, and vaccine responsiveness. Conversely, undernutrition, parasitism, and micronutrient deficiency can blunt immune responses and increase susceptibility to severe PPR, particularly in young or pregnant animals. In smallholder systems, therefore, nutritional support should be considered part of disease prevention rather than merely a production issue.

Probiotics, prebiotics, and immunobiotic feed supplements have been proposed as adjunctive immunomodulatory strategies, based on evidence from other host–pathogen systems that selected microbial strains can influence mucosal immunity and antiviral responses [57,58]. However, no study has directly tested whether such supplementation affects PPRV infection, clinical severity, or vaccine responsiveness in goats. We therefore present immunobiotic supplementation as a hypothesis-generating research direction (Section 6.2) rather than as an established or currently recommended component of PPR control.

Although immunocompetence is influenced by environmental and management factors, growing evidence indicates that it also has a heritable component. Variability in susceptibility to PPR, vaccine responsiveness, inflammatory regulation, and recovery following infection may therefore arise partly from host genetic differences. Understanding these genetic determinants is essential for explaining breed-specific variation in disease outcome and for developing complementary breeding strategies that can enhance long-term resilience alongside vaccination-based control.

## 5. Host Genetics for PPR Control

Genetic resistance to PPR in goats is best viewed as a complex host phenotype shaped by multiple genes involved in antiviral sensing, innate and adaptive immune regulation, inflammatory control, epithelial barrier function, and tissue repair. In practice, resilience may be expressed not only as reduced susceptibility to infection, but also as lower viral replication, milder clinical disease, reduced mortality, faster recovery, or more robust vaccine responsiveness. At present, however, the molecular basis of resistance to PPR virus (PPRV) in goats remains poorly defined, and no major resistance locus has yet been validated. Current evidence is therefore indirect and derives largely from three complementary sources: (i) breed-level differences in field susceptibility or vaccine response, (ii) transcriptomic studies of host responses to PPRV infection, and (iii) comparative and whole-genome studies that identify immune-related genetic variation in goat populations [27,59,60,61,62]. These emerging datasets support the plausibility of host genetic resistance or resilience to PPR, but they do not yet establish causality. Consequently, the immediate value of immunogenomics in PPR lies in identifying biologically plausible candidate pathways and generating testable markers for future association and breeding studies.

For clarity, we distinguish several related but biologically distinct concepts that are frequently conflated in the PPR literature. *Susceptibility* refers to the baseline probability that exposure to PPRV results in infection or clinical disease; it is the aggregate outcome of resistance and tolerance mechanisms and is typically measured indirectly through infection or attack rates in field studies. *Resistance to infection* refers to a host’s capacity to prevent or substantially limit viral establishment and replication following exposure, and is ideally demonstrated by lower infection rates or viral loads under standardized challenge. *Disease tolerance* describes the capacity to limit the clinical or pathological impact of a given pathogen burden without necessarily reducing that burden, whereas *resilience* is a broader concept describing the capacity of an animal or population to maintain acceptable health, growth, or production despite infection or other perturbations. *Reduced clinical severity* and improved survival are outcome measures that can result from resistance, tolerance, or resilience mechanisms acting individually or in combination, and on their own cannot distinguish among them. *Vaccine responsiveness* reflects the adaptive immune system’s capacity to mount protective immunity following vaccination and is conceptually distinct from innate resistance to natural infection, although the two may share underlying immunogenetic pathways. Most of the evidence reviewed in this section (breed-level differences in field morbidity, transcriptomic responses to infection, and comparative genomic variation) speaks primarily to resilience, tolerance, and vaccine responsiveness rather than to demonstrated resistance to infection in the strict sense, and we use these terms accordingly throughout this section.

### 5.1. Black Bengal Goat Genomic Resources and Their Relevance to PPR Research

The Black Bengal goat is an important genetic resource for studying host resilience in an endemic PPR setting. Several genomic resources are now available for the Black Bengal goat, including draft genome assemblies, whole-genome resequencing datasets, and catalogues of single-nucleotide polymorphisms (SNPs) and small insertions/deletions (indels) [17,27,59,60]. These datasets do not demonstrate resistance to PPR per se, but they provide the foundational infrastructure required to investigate disease-associated variation in locally adapted goat populations.

The first draft genome assembly of the Black Bengal goat reported an assembly size of approximately 3.04 Gb and annotated 26,458 gene models, with a BUSCO completeness of 82.5% [52]. Independent whole-genome analyses also identified large numbers of exonic variants, genome-wide SNPs, and indels, including variation in genes previously associated with stress response, apoptosis, muscle biology, and other production-related traits [54]. Although these studies were not designed to map PPR resistance, they are nevertheless valuable because they establish reference genomic resources for downstream genome-wide association studies (GWAS), selective sweep analyses, expression quantitative trait locus mapping, and comparative immunogenomic analyses [61]. For a disease such as PPR, which is likely influenced by many loci of small to moderate effect, such population-scale genomic resources are a prerequisite for moving from anecdotal breed resilience to evidence-based marker discovery.

More broadly, goat population genomics has demonstrated substantial adaptive diversity among indigenous breeds exposed to different climates, pathogens, and management systems. For example, resequencing studies in African goats have identified signatures of selection in loci linked to immunity, environmental adaptation, reproduction, and metabolism, highlighting the potential of locally adapted breeds as reservoirs of disease-relevant variation [62]. In this context, the Black Bengal goat should be regarded not as a proven “PPR-resistant” genotype, but as a biologically and epidemiologically important population in which resistance- or resilience-associated variants can be systematically investigated.

In summary (Table 2), what is currently known about Black Bengal goats in relation to PPR is largely descriptive: the breed is reported to experience lower apparent morbidity and mortality under some field conditions, and a growing set of genomic resources now exists for it. What remains unknown is whether any of this apparent resilience has a genetic basis, since no study has yet linked Black Bengal genomic variation to PPR infection status, viral load, clinical severity, or vaccine response. Research designs that could directly test PPR resilience in this breed include: (i) prospective cohort studies that genotype animals prior to natural PPRV exposure and relate genotype to subsequent infection status, viral load, and clinical outcome; (ii) case–control genome-wide association studies comparing severely affected and mildly affected or unaffected animals from the same outbreak and management context; (iii) controlled experimental challenge studies, where ethically and logistically feasible, comparing Black Bengal goats with other breeds under standardized infectious dose and husbandry conditions; and (iv) vaccine-response phenotyping studies relating genomic variation to neutralizing antibody titers and cell-mediated immune responses following standardized vaccination. Until such designs are implemented, claims of genetic resistance or resilience in this breed should remain provisional.

### 5.2. PPRV Genomic Features Relevant to Host–Virus Interactions

PPRV is an enveloped, non-segmented, negative-sense single-stranded RNA virus of the genus Morbillivirus in the family Paramyxoviridae. Its genome is 15,948 nucleotides in length and follows the canonical morbillivirus gene order 3′-N-P/C/V-M-F-H-L-5′, encoding six structural proteins—nucleoprotein (N), phosphoprotein (P), matrix protein (M), fusion protein (F), hemagglutinin protein (H), and large polymerase protein (L)—together with the accessory C and V proteins generated from the P gene by alternative expression strategies [4,63]. The H and F glycoproteins mediate receptor binding and membrane fusion, the N–P–L complex is responsible for genome encapsidation and replication, and the M protein coordinates virion assembly and budding. From the perspective of host resistance, the most relevant viral proteins are those involved in cell entry, tropism, and immune evasion.

As discussed in Section 3, PPRV enters immune cells primarily through signaling lymphocyte activation molecule (SLAM/CD150) and spreads to epithelial tissues via nectin-4. The viral V protein is a major antagonist of interferon induction and signaling, whereas the H and F glycoproteins are central to tropism, immunogenicity, and neutralization [64]. These features are directly relevant to genetic resistance because host polymorphisms in viral receptors, interferon signaling pathways, antigen processing, and inflammatory regulation could theoretically influence susceptibility, disease severity, or vaccine response. Although PPRV is serologically monotypic, it is genetically classified into four lineages (I–IV), with lineage IV now dominating across much of Asia, the Middle East, and increasingly Africa [4,40]. In Bangladesh, available molecular evidence indicates circulation of lineage IV viruses. Continued genomic surveillance of field strains remains important for tracing viral movement and contextualizing host–pathogen co-evolution, even though lineage classification alone does not explain differences in host resistance.

### 5.3. Molecular Genetics of Host–PPRV Interactions

#### 5.3.1. Pattern-Recognition Receptors

Because PPRV is an RNA virus that initially targets mucosal and lymphoid tissues, the most plausible host resistance pathways are those involved in viral sensing, interferon signaling, lymphocyte activation, cytokine regulation, and maintenance of epithelial integrity. Pattern-recognition receptors such as Toll-like receptors (TLRs), retinoic acid-inducible gene I (RIG-I)-like receptors, melanoma differentiation-associated protein 5 (MDA5), and downstream interferon regulatory factors (IRFs) are central to the early detection of viral RNA and the initiation of antiviral responses. In goats, intracellular TLRs, including TLR3, TLR7, and TLR8, are of particular interest because they recognize viral nucleic acids and may influence the strength and timing of innate immune activation. However, evidence directly linking polymorphisms in these receptors to PPR susceptibility remains limited.

One candidate frequently discussed in goat immunogenetics is TLR7, an endosomal receptor that senses single-stranded RNA viruses. Sequence characterization of caprine TLR7 has identified coding and untranslated-region polymorphisms, but no study has yet demonstrated a reproducible association between TLR7 genotype and resistance to PPR in goats [28]. Thus, TLR7 should currently be regarded as a biologically plausible candidate gene rather than a validated marker of PPR resistance. The same caution applies to other immune genes frequently highlighted in comparative studies, including interferon-related genes, cytokine regulators, and genes involved in leukocyte signaling and apoptosis.

#### 5.3.2. Transcriptomic Responses

Transcriptomic studies provide the strongest current molecular evidence that host responses to PPRV involve pathways relevant to genetic resistance. In goat peripheral blood mononuclear cells and other infection models, PPRV has been shown to alter the expression of genes involved in innate immunity, interferon signaling, apoptosis, inflammation, and antiviral defense [4,29]. These studies consistently support the concept that disease outcome is influenced by the host’s ability to mount a timely antiviral response while limiting excessive tissue-damaging inflammation [65].

Among the genes implicated in experimental systems, tripartite motif-containing protein 56 (TRIM56) is of particular interest because it has antiviral activity and participates in interferon-mediated restriction of RNA viruses. In goat cells, TRIM56 expression has been linked to suppression of PPRV replication, possibly through interactions with interferon regulatory pathways [29,30]. Similarly, dysregulation of transcription factors and immune signaling mediators—including members of the IRF family—has been observed following PPRV infection, further supporting a role for interferon-centric networks in host defense. However, transcriptomic induction of a gene during infection should not be equated with inherited resistance. Rather, such genes should be viewed as candidates for subsequent validation in genetic association, functional genomics, and gene-expression studies conducted in naturally exposed goat populations.

#### 5.3.3. Comparative Genomics and Immune-Gene Variation

Comparative genomics of domestic goats has also identified copy number variation and lineage-specific changes in immune-related genes that may be relevant to disease resilience. For example, comparisons between wild and domestic goats have reported copy number changes involving genes such as TRIM5, CFH, ABCC4, PRAME, CD163L1, and KIR3DL1, several of which are associated with antiviral defense, complement regulation, or immune cell function [31,32]. These findings collectively indicate that host immune responses to PPRV are shaped by multiple interacting pathways rather than individual genes, emphasizing the need for integrated immunogenomic studies that combine functional genomics with carefully phenotyped field populations.

#### 5.3.4. Future Immunogenomics

The current molecular evidence supports a working model in which genetic resistance or resilience to PPR is likely polygenic and mediated through pathways controlling early viral sensing, interferon signaling, lymphocyte function, inflammatory balance, and tissue recovery [30,31,32,65]. The most promising next step is therefore not to infer resistance from single candidate genes, but to combine genomic variation data with well-phenotyped field cohorts, vaccine-response studies, and transcriptomic or proteomic profiling in endemic goat populations.

### 5.4. Prospects of Genetic Control for PPR

The prospect of improving host genetic resistance to PPR through breeding is biologically plausible but remains at an early stage. Across livestock species, selection for disease resistance has generally been more successful when resistance is moderately heritable, measurable at scale, and linked to robust phenotypes or molecular markers [66]. For PPR in goats, these prerequisites are not yet fully in place. Nevertheless, several observations justify further investment in this area. Throughout this section, we use “indigenous” to refer to breeds that have developed through long-term natural and traditional selection within a given region (e.g., the Black Bengal and Assam Hill goat in South Asia), and “local” more broadly to refer to any breed or population currently raised in a given production system, regardless of its origin, including indigenous breeds as well as long-established exotic or crossbred populations. We do not intend these terms to be interchangeable: an indigenous breed is by definition local to its region of origin, but a local population is not necessarily indigenous. First, local and indigenous breeds often appear to differ in clinical susceptibility, survival, or vaccine responsiveness under field conditions. Observational studies suggest that some locally adapted breeds, including the Black Bengal and Assam Hill goat, may exhibit lower apparent disease severity or stronger vaccine-induced immune responses under endemic field conditions than certain exotic or crossbred populations. However, these differences have not yet been conclusively attributed to host genetic factors because environmental adaptation, management, nutrition, and previous pathogen exposure may also contribute substantially to the observed variation [27,67,68]. Second, the genomic resources now available for Black Bengal and other indigenous goats make it feasible to test these hypotheses more rigorously.

In practical terms, breeding for PPR resilience should initially focus on marker discovery and phenotype definition, not immediate selection. The most informative phenotypes are likely to include laboratory-confirmed infection status, survival or case fatality, clinical severity scores, viral load, duration of shedding, and standardized vaccine-response traits such as seroconversion and neutralizing antibody kinetics [62,69]. Integrating these phenotypes with GWAS, regional heritability mapping, selective sweep analyses, and expression studies in endemic goat populations would allow candidate loci to be prioritized for validation. Such work will require larger sample sizes, careful control of confounding by vaccination and management, and longitudinal designs that distinguish resistance to infection from tolerance of disease.

In the longer term, validated markers could support genomic selection or marker-assisted breeding for PPR resilience, especially in locally adapted breeds maintained in endemic regions. Gene editing approaches such as CRISPR-Cas9 systems are conceptually possible [70], but at present they remain premature for PPR because the causal genes and target variants have not been established, and regulatory, ethical, and deployment constraints remain substantial. Therefore, the most realistic near-term pathway is to use immunogenomics to identify resistance-associated markers and integrate them into conventional breeding and health-management strategies rather than to propose immediate genome editing for PPR control. Taken together, the available evidence suggests that host genetics is likely to contribute to variation in PPR susceptibility and outcome in goats, but the field is still in a discovery phase [4,71]. For endemic production systems such as those based on the Black Bengal goat, the priority should be to combine epidemiological surveillance, vaccine-response phenotyping, and population genomics to determine whether heritable resistance or resilience to PPR can be quantified and harnessed for sustainable disease control.

## 6. Discussion and Recommendations

### 6.1. Integrating Epidemiology, Immunocompetence, and Host Genetics

**Figure 1:**
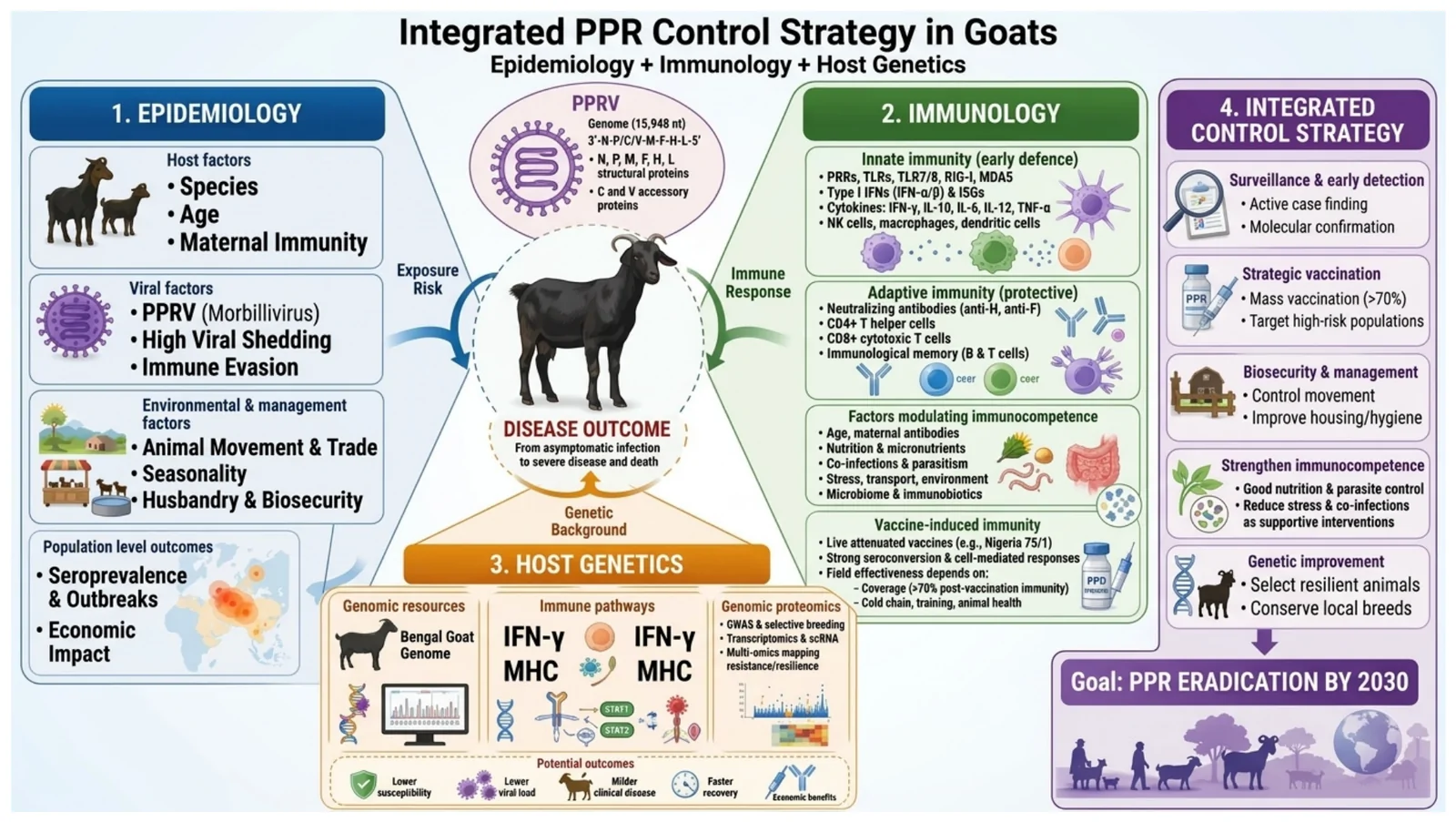
Integrated conceptual framework illustrating the interactions among epidemiology, immunology, and host genetics in determining PPR transmission, disease outcome, and control. Epidemiological drivers (host, virus, and environment) determine exposure risk, while host immune competence and genetic variation influence susceptibility, vaccine responses, viral clearance, and clinical severity. Together, these interconnected components determine herd immunity and provide the foundation for integrated surveillance, vaccination, breeding, and management strategies supporting the global eradication of PPR.

A central concept emerging from this review is that epidemiology, immunocompetence, and host genetics should not be viewed as independent determinants of PPR but rather as interconnected components of a dynamic disease system (Figure 1). Epidemiological factors determine the likelihood and frequency of viral exposure, whereas immunocompetence governs the efficiency of antiviral responses and the magnitude and durability of vaccine-induced protection. Host genetic variation further modifies these immune responses by influencing innate viral recognition, cytokine regulation, lymphocyte activation, and tissue repair. Consequently, the same viral challenge may result in markedly different clinical outcomes among animals exposed under similar environmental conditions.

This integrated framework also helps explain why vaccination alone does not always achieve optimal population immunity in endemic production systems. Vaccine effectiveness is influenced not only by vaccine quality and coverage but also by host nutritional status, maternal antibodies, concurrent infections, stress, genetic background, and management practices [51,54,67]. Therefore, sustainable PPR control requires coordinated implementation of epidemiological surveillance, strategic vaccination, improved husbandry, and host-directed approaches aimed at enhancing immune competence and identifying genetically resilient animals. Integrating these disciplines provides a stronger scientific foundation for achieving the objectives of the FAO–WOAH Global Eradication Programme [10].

From an epidemiological perspective, PPR outcome is shaped by the interaction of the host, virus, and environment. Transmission is influenced not only by viral circulation and animal movement, but also by flock demography, herd immunity, husbandry practices, nutrition, co-infections, and the strength of veterinary services [41,72]. Consequently, it requires coordinated surveillance, rapid outbreak reporting, movement management, biosecurity, and sustained vaccination programmes designed to achieve and maintain sufficient population immunity. Although live attenuated vaccines provide excellent protection against PPRV, effectiveness depends on achieving sufficient vaccination coverage and maintaining population immunity through sustained surveillance and repeated vaccination of susceptible cohorts [53,64,73,74]. Field experiences from the PPR Global Eradication Programme indicate that post-vaccination immunity levels of approximately 70% or higher are needed to interrupt transmission in high-risk populations, although the exact threshold will vary with local epidemiology, population turnover, and campaign performance [33,43].

Host immunocompetence is equally central to PPR prevention. Protective immunity against PPRV depends on effective innate antiviral sensing, timely interferon responses, competent T- and B-cell activation, and durable vaccine-induced memory [47,64]. In practice, however, immunocompetence is not determined by vaccination alone. Host immunocompetence is influenced by age, maternal antibodies, nutritional status, stress, parasitism, concurrent infections, microbiome composition, and host genetics [44]. Therefore, improving the health and resilience of goats through better feeding, parasite control, reduction in chronic stress, and stronger preventive health management should be viewed as complementary to vaccination rather than separate from it [73,75]. Such measures are especially relevant in low-input systems, where nutritional and infectious stressors may blunt vaccine responses and increase the severity of PPR outbreaks.

The role of host genetics in PPR remains promising but insufficiently resolved. Breed-level differences in apparent susceptibility, survival, or vaccine responses suggest that inherited factors may contribute to disease outcome, and genomic resources from indigenous breeds such as the Black Bengal goat provide an important platform for testing this hypothesis [32,61,69,76]. However, there is still no validated major resistance locus for PPR in goats, and the current evidence points more strongly to a polygenic resilience phenotype involving antiviral sensing, interferon signaling, inflammatory regulation, and recovery from tissue injury rather than to simple Mendelian resistance. For this reason, the immediate priority is not to claim genetic resistance prematurely, but to define robust phenotypes and identify reproducible molecular markers associated with reduced clinical severity, lower viral load, shorter shedding, improved survival, or stronger vaccine responses.

### 6.2. Research Priorities

Several research priorities emerge from this synthesis. First, longitudinal field studies are needed in endemic goat populations to link laboratory-confirmed PPR outcomes with breed, pedigree, vaccination status, and management variables. Second, integrated immunogenomic studies should combine genome-wide genotyping or resequencing with transcriptomic, serological, and clinical phenotyping in naturally exposed or experimentally vaccinated goats. Time-resolved profiling of respiratory and intestinal mucosa, draining lymphoid tissues, and peripheral blood mononuclear cells during early infection and after vaccination would be particularly valuable for identifying candidate pathways associated with protection or pathology. Third, vaccine evaluation in local breeds should move beyond simple seroconversion and include standardized measures of neutralizing antibody kinetics, cell-mediated immunity, duration of protection, and field effectiveness under real production conditions. This is especially relevant for Black Bengal goats and other locally adapted breeds in South Asia, where endemic exposure, nutritional constraints, and management heterogeneity may shape immune performance.

The potential role of immunobiotics, probiotics, or other nutritional immunomodulators also warrants careful investigation, but current evidence should be interpreted cautiously. Experimental studies in other livestock species indicate that selected probiotic strains can modulate mucosal immunity and antiviral responses, suggesting a potential adjunctive role in enhancing overall immune resilience [77,78]. However, there is currently no direct evidence that goat-derived probiotics prevent PPRV infection or materially enhance protection against PPR under field conditions. Accordingly, immunobiotics should be considered a research priority for supportive immune modulation, not a substitute for vaccination or core biosecurity measures.

Finally, advances in genomics, systems immunology, and reproductive biotechnology offer important long-term opportunities, but their application to PPR control must remain evidence-driven [79,80,81]. Genome editing approaches such as CRISPR/Cas are conceptually attractive [70], yet they are premature for PPR because the causal variants underlying resistance or resilience have not been established. In the near term, a more realistic pathway is to use genomics to support marker discovery, genomic selection, and conservation of locally adapted breeds with favorable health and production traits [71]. For Black Bengal goats and other indigenous populations, this approach could ultimately complement vaccination-based control by strengthening the biological resilience of herds in endemic environments.

## 7. Conclusions

This review argues that sustainable control of PPR requires a shift from viewing vaccination as a stand-alone intervention to understanding PPR as a biologically and epidemiologically complex disease shaped by the interaction of viral, host, and environmental factors. Rather than representing independent research fields, epidemiology, host immunocompetence, and genetics together define the biological and ecological framework within which PPR persists or is eliminated. Epidemiology identifies where transmission occurs and which populations are at greatest risk; immunology determines the effectiveness of natural and vaccine-induced protection; and host genetics contributes to variation in susceptibility, disease severity, and vaccine responsiveness, with current evidence pointing more toward heritable differences in resilience, tolerance, and vaccine responsiveness than toward proven resistance to infection. Future PPR research should increasingly integrate these disciplines through longitudinal field epidemiology, systems immunology, multi-omics, and quantitative genetics. Such interdisciplinary approaches will improve understanding of host–virus interactions and support the development of more sustainable, evidence-based PPR prevention and control strategies, particularly for locally adapted goat populations in endemic regions, and eventual global eradication.

## Figures and Tables

**Table 1:** Representative epidemiological, immunological, and genetic studies discussed in this review, illustrating study design, key findings, and principal limitations to aid critical comparison across the evidence base. Bracketed numbers refer to the reference list.

Reference(s)	Domain	Study Design/Population	Key Finding	Principal Limitation/Caveat
[12]	Epidemiology	Systematic review and meta-analysis, sheep and goats, Africa and Asia, 1969–2018	Pooled seroprevalence of approximately 31% in Bangladeshi goats, indicating sustained viral circulation	Pools studies using heterogeneous diagnostic assays and vaccination histories, limiting direct comparability across settings
[21]	Epidemiology	Field epidemiological and spatial-cluster study, Mymensingh, Bangladesh	Identified risk factors and space-time clustering of PPR outbreaks	Single-district study; generalizability to other production systems and regions is uncertain
[22]	Epidemiology/breed susceptibility	Field observational comparison across goat breeds, Bangladesh	Lower apparent morbidity and mortality reported in Black Bengal goats than in exotic or crossbred populations	Confounded by management, nutrition, and vaccination history; does not establish genetic causation
[23]	Immunology (innate)	Experimental PPRV infection with cytokine profiling	Documented early induction of IFN-β, IL-6, and related cytokines during acute infection	Single-strain experimental infection; timing and magnitude may not generalize to field strain diversity
[24]	Immunology (vaccine)	Experimental comparison of Nigeria 75/1 and Sungri/96 vaccine strains	Both classical live attenuated strains induce durable, cross-lineage protective immunity	Controlled experimental conditions; field cold-chain, nutritional, and management variables not captured
[25,26]	Immunology (field vaccine)	Field and experimental vaccine trials, Bangladesh	Conventional and thermostable vaccine formulations both induce seroconversion in goats	Seroconversion was measured rather than field protection; storage and handling variability not fully controlled
[27]	Genetics (genomic resource)	Whole-genome sequencing and assembly, Black Bengal goat	Draft assembly of approximately 3.04 Gb; 26,458 annotated gene models; 82.5% BUSCO completeness	Reference genome only; not linked to any PPR challenge or field-phenotype data
[28]	Genetics (candidate gene)	Sequence characterization of caprine TLR7	Coding and untranslated-region polymorphisms identified in TLR7	No reproducible association with PPR resistance has yet been demonstrated
[29,30]	Genetics (transcriptomics)	Transcriptomic profiling of PPRV- and vaccine-infected goat PBMCs	TRIM56 induction and IRF-family dysregulation associated with the antiviral response	Transcriptional induction during infection does not establish inherited resistance; requires genetic-association validation
[31,32]	Genetics (comparative genomics)	Comparative whole-genome/copy-number-variation analysis, wild vs. domestic goats	Copy-number variation identified in TRIM5, CFH, ABCC4, PRAME, CD163L1, and KIR3DL1	Not linked to PPR phenotypes in the source studies; relevance to PPR susceptibility is inferred, not tested
[33]	Vaccination programme	Field post-vaccination sero-monitoring, Karnataka, India	Documented progress toward PPR eradication through post-vaccination sero-monitoring	Serological monitoring alone does not fully capture clinical protection or on-the-ground field effectiveness
[34]	Vaccine platforms (experimental)	Review of capripoxvirus-vectored vaccine platforms	DIVA-compatible vector-based platforms are conceptually feasible and immunogenic in laboratory studies	Experimental/pre-clinical stage only; not yet licensed or deployed in routine field vaccination campaigns

**Table 2:** Black Bengal goat genomic resources, their relevance to PPR research, and current limitations. Bracketed numbers refer to the reference list.

Resource	Description	Relevance to PPR Research	Limitation
Draft genome assembly [27]	~3.04 Gb assembly; 26,458 annotated gene models; 82.5% BUSCO completeness	Reference genome enabling variant calling, GWAS, and eQTL mapping	Not linked to any PPR challenge or field-phenotype data
Complete mitochondrial genome [59]	Full mitogenome sequence of the breed	Useful for maternal lineage and phylogeographic studies	Mitochondrial markers do not capture nuclear immune-pathway variation relevant to PPR resistance
Whole-genome resequencing, Savar farm population [60]	Genome-wide SNP/indel catalogue from a defined farm population	Basis for future GWAS and selective-sweep analyses in a defined population	Single-farm sampling; PPR exposure and vaccination history of sequenced animals not characterized
General goat genomic-resource reviews [17]	Broader review of caprine genomic tools and economically important gene targets	Contextualizes Black Bengal-specific resources within wider caprine genomics	Not specific to Black Bengal goats or to PPR; focused on economic traits rather than disease resistance
PPR-specific virological/pathological characterization [5]	Documents PPRV pathogenesis and tissue pathology directly in Black Bengal goats	Provides a breed-specific clinical and pathological baseline for future genetic-association work	Small-scale natural-infection case series; no genetic variant data or association testing performed

## Data Availability

No new data were created or analyzed in this study. Data sharing is not applicable for this article.

## Supplementary Materials

The following supporting information can be downloaded at https://www.mdpi.com/article/10.3390/v18091007/s1, Table S1. Literature search strategy used for evidence synthesis, including eligibility scope, databases searched, execution dates, and records retrieved per concept block.
