# Supplementary material for: Integrating Epidemiology, Immunology, and Host Genetics for Controlling Peste des Petits Ruminants in Goats
Source: Viruses. 2026 Sep 13;18(9):1007. doi: 10.3390/v18091007 (PMC13612065; doi:10.3390/v18091007)
Supplement: Supplementary file 1 [file viruses-18-01007-s001.zip › viruses-4511151-supplementary.pdf]

Supplementary Table S1. Literature search strategy used for evidence synthesis, including eligibility scope, databases searched, execution dates, and records retrieved per concept block.

Concept block	Representative search terms and combinations*	Database(s) searched	Search execution date(s)	Records retrieved
1. Epidemiology of PPR	(“Peste des petits ruminants” OR PPR OR PPRV) AND (epidemiology OR prevalence OR incidence OR outbreak OR transmission OR endemic)	PubMed (MEDLINE)	August 26, 2026	1347
2. Risk factors and transmission dynamics	(“Peste des petits ruminants” OR PPRV) AND (“risk factor*” OR transmission OR susceptibility OR morbidity OR mortality OR “animal movement” OR biosecurity OR husbandry)	PubMed (MEDLINE)	August 26, 2026	634
3. Disease frequency, distribution, and surveillance	(“Peste des petits ruminants” OR PPRV) AND (prevalence OR seroprevalence OR surveillance OR distribution OR “spatial epidemiology” OR “molecular epidemiology”)	PubMed (MEDLINE)	August 26, 2026	592
4. Vaccines and vaccination	(“Peste des petits ruminants” OR PPRV) AND (vaccine* OR vaccination OR immunization OR “live attenuated vaccine” OR DIVA OR thermostable OR recombinant vaccine)	PubMed (MEDLINE)	August 26, 2026	549
5. Immunocompetence and vaccine-induced immunity	(“Peste des petits ruminants” OR PPRV) AND (immunocompetence OR immunity OR “immune response” OR “innate immunity” OR “adaptive immunity” OR cytokine* OR interferon OR transcriptome OR immunogenomics)	PubMed (MEDLINE)	August 26, 2026	427
6. Black Bengal goats and caprine genomics	(“Black Bengal goat” OR “Capra hircus”) AND (genome OR genomics OR “whole genome sequencing” OR SNP OR GWAS OR transcriptome OR “genetic diversity”)	PubMed (MEDLINE)	August 26, 2026	398
7. Host–PPRV interactions and molecular mechanisms	(“Peste des petits ruminants virus” OR PPRV) AND (“host-pathogen interaction” OR transcriptomics OR RNA-seq OR proteomics OR “gene expression” OR “immune signaling” OR TLR OR interferon OR TRIM56)	PubMed (MEDLINE)	August 26, 2026	89
8. Genetic resistance, resilience, and breeding	(“Peste des petits ruminants” OR PPRV) AND (“genetic resistance” OR resilience OR susceptibility OR “marker-assisted selection” OR “genomic selection” OR CRISPR OR “genome editing”)	PubMed (MEDLINE)	August 26, 2026	88
9. Surveillance, policy, and eradication	(“Peste des petits ruminants” OR PPRV) AND (WOAH OR FAO OR surveillance OR eradication OR policy OR regulation OR “Global Eradication Programme” OR “transboundary animal disease”)	PubMed (MEDLINE)	August 26, 2026	698

*Searches combined Medical Subject Headings (MeSH), where available, and free-text keywords, and were supplemented by backward citation tracking of relevant publications and authoritative documents from the World Organisation for Animal Health (WOAH) and the Food and Agriculture Organization of the United Nations (FAO). The literature search was conducted from January 01, 2020 through August 26, 2026.

Full inclusion and exclusion criteria, the evidentiary hierarchy applied when direct goat-specific evidence was limited, and the screening process are described in Section 2 (Search Strategy and Evidence Synthesis) of the main text.

Records retrieved reflect the number of unique records returned by the concept-block search string prior to de-duplication across blocks and removal of records excluded during title/abstract or full-text screening.
